# Protein Arginine Methyltransferases (PRMTs): Promising Targets for the Treatment of Pulmonary Disorders

**DOI:** 10.3390/ijms131012383

**Published:** 2012-09-27

**Authors:** Dariusz Zakrzewicz, Anna Zakrzewicz, Klaus T. Preissner, Philipp Markart, Malgorzata Wygrecka

**Affiliations:** 1 Department of Biochemistry, Faculty of Medicine, University of Giessen Lung Center, Friedrichstrasse 24, 35392 Giessen, Germany; 2 Laboratory of Experimental Surgery, Department of General and Thoracic Surgery, Justus Liebig-University Giessen, Rudolf-Buchheim-Str. 7, D-35385 Giessen, Germany; E-Mail: anna.zakrzewicz@chiru.med.uni-giessen.de; 3 Department of Biochemistry, Faculty of Medicine, Universities of Giessen and Marburg Lung Center (UGMLC), Member of the German Center for Lung Research, Giessen, Germany; E-Mails: klaus.t.preissner@biochemie.med.uni-giessen.de (K.T.P.); malgorzata.wygrecka@innere.med.uni-giessen.de (M.W.); 4 Department of Internal Medicine, Faculty of Medicine, Universities of Giessen and Marburg Lung Center (UGMLC), Member of the German Center for Lung Research, Giessen, Germany; E-Mail: philipp.markart@innere.med.uni-giessen.de

**Keywords:** protein arginine methylation, PRMT, chronic lung disease

## Abstract

Protein arginine methylation is a novel posttranslational modification that plays a pivotal role in a variety of intracellular events, such as signal transduction, protein-protein interaction and transcriptional regulation, either by the direct regulation of protein function or by metabolic products originating from protein arginine methylation that influence nitric oxide (NO)-dependent processes. A growing body of evidence suggests that both mechanisms are implicated in cardiovascular and pulmonary diseases. This review will present and discuss recent research on PRMTs and the methylation of non-histone proteins and its consequences for the pathogenesis of various lung disorders, including lung cancer, pulmonary fibrosis, pulmonary hypertension, chronic obstructive pulmonary disease and asthma. This article will also highlight novel directions for possible future investigations to evaluate the functional contribution of arginine methylation in lung homeostasis and disease.

## 1. Introduction

Pulmonary diseases (PD), including lung cancer, idiopathic pulmonary fibrosis (IPF), pulmonary hypertension (PH), chronic obstructive pulmonary disease (COPD) and asthma are the second leading cause of death worldwide. Their rate of incidence is on the increase and PD are usually associated with a high socio-economic burden [1,2]. Despite substantial progress in understanding the epidemiology and pathophysiology, we still lack efficient pharmacological therapeutic options for many PD, particularly in regard to improving mortality rates. As lung transplantation is an option for only a limited number of patients with end-stage disease [3–6], there is an urgent need to identify novel, more effective therapies for PD.

Post-translational modifications of proteins play an important role in signal transduction allowing cells to respond to changes and events occurring in extracellular milieu. Although the role of acetylation and phosphorylation has already been extensively discussed in the context of PD [7–10], the importance of other types of protein modifications, including ubiquitination and methylation has now begun to be recognized [11,12]. Protein arginine methylation represents a novel protein modification that has been implicated in a number of cellular processes including transduction of intracellular signaling [13], DNA repair [14–16], RNA processing [17,18], protein-protein interaction and regulation of gene expression [19,20], thereby controlling cell differentiation, proliferation, migration and apoptosis [21,22]. Thus, any alteration of intracellular protein methylation may disturb cellular homeostasis and consequently lead to various, uncontrolled, pathological events observed in PD.

## 2. Protein Arginine Methylation

**Figure 1:**
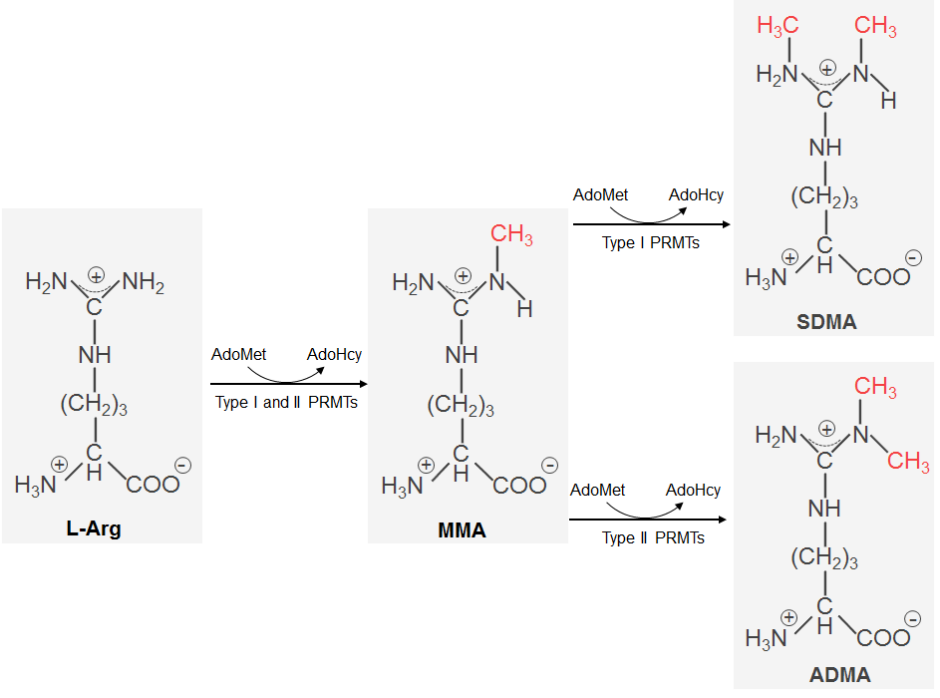
The mechanism of protein arginine methylation in mammalian cells. L-Arg can be monomethylated on a guanidino nitrogen atom by all protein arginine methyltransferases (PRMTs). Type I PRMTs catalyze the formation of asymmetric dimethylarginine, while type II PRMTs generate symmetric dimmethylarginine. The donor of methyl groups is *S*-adenosylmethionine (AdoMet), which is further converted to *S*-adenosylhomocysteine (AdoHcy).

Protein arginine methylation was initially discovered in the calf thymus over 40 years ago by W. K. Paik [23]. Since then, arginine methylation has been extensively studied, demonstrating its great importance in the regulation of protein functions in a variety of fundamental cellular processes. The methylation of protein arginine residues is catalyzed by a family of intracellular enzymes termed protein arginine methyltransferases (PRMT) [11,24]. They catalyze the addition of one or two methyl groups to the guanidino nitrogen atoms of arginine resulting in either ω-NG-monomethylarginine (MMA), ω-NG, NG-asymmetric (ADMA) or ω-NG, N′G-symmetric dimethylarginine (SDMA). In humans, PRMTs have been classified into type I and type II, depending on their specific catalytic activities. Both types of enzymes first catalyze the formation of MMA as an intermediate. In a second step, type I enzymes (PRMT1 [25], PRMT3 [26], CARM1/PRMT4 [19], PRMT6 [27] and PRMT8 [28,29]) lead to the formation of ADMA, whereas the type II enzymes (PRMT5 [30,31] and PRMT7 [32,33]) produce SDMA (Figure 1). Of note is that PRMT2 was previously classified into type I enzymes [34] but its methyltransferase activity has just recently been characterized *in vitro* [35]. After breakdown of mono- and dimethylated intracellular proteins, free MMA, ADMA and SDMA can be released into cells.

Thus, methylation of arginine residues within proteins by PRMTs and the subsequent proteolysis of these arginine-methylated proteins by proteasome and autophagy pathways represent the major source of free intracellular methylarginine [36–38], since there is currently no evidence that free L-arginine (L-Arg) can be methylated [39].

Free cellular MMA and ADMA, but not SDMA, can be intracellularly degraded to citrulline and mono- or dimethylamines, respectively, by two dimethylarginine dimethylaminohydrolases (DDAH): DDAH1 and DDAH2 [11,36]. Alternatively, ADMA can be converted to α-keto valeric acid by alanine:glyoxylate aminotransferase 2 [40], and SDMA may be catabolized *in vivo* when injected intraperitonelly into rats, although the enzymes involved have not been identified thus far [41].

## 3. Protein Arginine Methyltransferases in Pulmonary Disorders

### 3.1. Lung Cancer

Lung cancer is the leading cause of cancer-related death worldwide. The prognosis of lung cancer is poor due to the fact that this disease can be symptomless in the early stage; therefore, most lung carcinomas are diagnosed at an advanced stage when distant metastases are already present. Current standard therapies include surgical resection, platinum-based doublet chemotherapy and radiation therapy alone or in combination. However, these therapies rarely cure the disease and the overall 5-year survival rate is still only 5%–15% [42–44]. Therefore, searching for new therapeutic agents and exploring novel intervention targets might provide more clinical benefits and indicate better outcomes in lung cancer therapy.

Based on histologic appearance and presumed cellular origin, lung cancer can be divided into two main classes. Small cell lung cancer (SCLC) is of neuroendocrine origin, while non-small cell lung cancer (NSCLC) is predominantly epithelial. NSCLC, which accounts for approximately 75% of all lung cancers, is divided further into adenocarcinoma, squamous cell carcinoma (SCC), and large cell carcinoma histologies [44].

A growing body of evidence suggests that PRMTs are involved in human carcinogenesis, including lung cancer. Similarly to previously published studies on breast, colon and bladder cancers [45,46], elevated PRMT1 and PRMT6 expression has recently been found in various types of lung cancer including SCLC and NSCLC [47]. As PRMT1 is a major type I PRMT, it is not surprising that its enhanced expression is mirrored by increased ADMA content in systemic circulation in lung cancer patients as compared to nontumor control subjects [47]. ADMA may control pulmonary cell behavior either via direct effects on gene expression and protein function [48] or via inhibition of nitric oxide synthase (NOS), which consequently leads to alterations in NO generation [49]. Overall, the role of ADMA in lung cancer biology remains elusive and further studies are needed to fully decipher the mechanism of its action in these pathological conditions.

In contrast, contribution of PRMTs to the pathogenesis of lung cancer is well recognized and confirmed in numerous *in vitro* studies. siRNA-mediated knockdown of PRMT1 and PRMT6 was found to lead to suppression of lung cancer cell growth, most probably by influencing G_1_-S transition in the cell cycle. Importantly, expression profile analysis of PRMT1 and PRMT6-depleted cells indicated that PRMT1 and PRMT6 operate within many cellular pathways, supporting their regulatory role in the cell cycle, RNA processing and chromatin modification, processes fundamentally important for cancer cell proliferation [47]. This is in line with previous studies using PRMT1-deficient mice, which demonstrated that the loss of PRMT1 in embryonic fibroblasts (MEFs) leads to spontaneous DNA damage, delay in cell cycle progression, and reduction of the cell growth [22]. Similarly to PRMT1, knockdown of PRMT6 inhibited estrogen-stimulated proliferation of breast cancer cells [50] and impaired cell migration and invasion of U2OS cells [51]. Moreover, reduced PRMT6 expression was associated with better overall relapse-free and distant metastasis-free survival in breast cancer patients with the estrogen receptor (ER (ESR1))-positive invasive ductile carcinoma, supporting the notion that PRMT6-dependent transcription and alternative splicing may also be involved in lung cancer pathophysiology [52].

There is no direct evidence for dysregulation of other PRMTs, such as PRMT2, PRMT4 and PRMT5 in lung tumorigenesis, although these molecules participate in the pathogenesis of other types of human cancer. For instance, PRMT2 and its splice variants were found to play a role in the progression of breast cancer by modulation of promoter activities of the ERα-targeted genes thereby controlling cancer cell proliferation [53].

PRMT4 overexpression has been demonstrated in grade-III breast cancers and prostate adenocarcinomas [54–57]. In human breast and prostate cancer cells, CARM1/PRMT4 knockdown resulted in the inhibition of cell proliferation and cell cycle progression and in the enhancement of cell apoptosis [55,56]. In colorectal cancer cells, CARM1/PRMT4 was reported to be an important positive regulator of Wnt/β-catenin-dependent signaling [58], a developmentally active pathway, well investigated in lung cancer biology [59,60]. This all indicates that increased PRMT4 expression may lead to pathological changes observed in tumorigenesis. However, in contrast to those studies, O’Brien *et al.* suggested that PRMT4 is required for proper differentiation of alveolar cells and that overexpression of PRMT4 rather inhibits than potentiates pulmonary epithelial cell proliferation during lung development [61]. This opposite effect of PRMT4 overexpression on lung alveolar cell growth might be explained by distinct gene expression profiles and by the presence or absence of different PRMT4 protein targets in alveolar versus cancer cells. Additionally, it has to be kept in mind that as PRMT4 is highly expressed in the lung tissue as compared to other organs, this suggests an important role of this molecule in maintenance of lung homeostasis [62,63]. Thus, abundant pulmonary PRMT4 expression together with its ability to control cell proliferation makes PRMT4 a potential target for further investigations on lung cancer development and progression.

Uncontrolled cell growth in human tissues is regulated by another member of the PRMT family, PRMT5. It is a molecule that may control proliferation of cancer cells by a mechanism involving HIF-1α activation or direct methylation of E2F-1 transcription factor [64,65]. In addition, PRMT5 is highly expressed in human breast cancer [66]. Coexpression of PRMT5 with programmed cell death 4 (PDCD4) influences tumor suppressor properties of PDCD4 resulting in accelerated tumor growth in a murine orthotopic model of breast cancer. In breast cancer patients whose tumors contain high level of PRMT5, elevated PDCD4 expression correlates with a worse outcome [66]. PRMT5 has also been implicated in tumorigenesis by its interaction with p53 protein [67], the most frequently inactivated gene in human cancers [68]. p53 can regulate apoptosis, cell cycle arrest and senescence, and re-activation of p53 may be a plausible target for cancer therapy [68–71]. It has recently been reported that DNA damage induces PRMT5-dependent p53 arginine methylation, an event that changes the biochemical properties and functional outcome of the p53 response. This response involves the activation of target genes, such as cyclin dependent kinase inhibitor p21, NOXA, APAF1 and PUMA, which are important in regulating p53-dependent growth arrest [71]. Hence, PRMT5 depletion in human osteosarcoma U2OS cells was found to induce p53-dependent apoptosis [67]. Thus, regulation of arginine metylation status in p53 may provide new therapeutical options for the treatment of various types of lung cancer [72].

Collectively, the findings mentioned above provide evidence that links dysregulated protein arginine methylation and aberrant PRMT expression/activity with oncogenic events observed during lung cancer development and progression. In addition, current results indicate that PRMT1 and PRMT6 are involved in lung tumorigenesis. However, further studies are needed to fully decipher the molecular mechanisms of their action.

### 3.2. Pulmonary Fibrosis

In contrast to the well-established role of PRMTs in cancer cell biology, studies of protein arginine methylation in IPF are limited. Accumulating evidence suggests however, that arginine methylation may be involved in the progression of IPF, a chronic, irreversible and fatal lung disease of unknown etiology [6,73]. The main features of IPF are alveolar cell apoptosis, proliferation of lung fibroblasts/myofibroblasts and extracellular matrix protein deposition in the lung interstitium [6,74]. The hallmark lesions of IPF are fibroblast foci occurring in subepithelial layers adjacent to areas of alveolar epithelial cell injury. Subepithelial localization of fibroblast foci strongly suggests that altered epithelial-mesenchymal crosstalk contributes to the pathobiology of IPF [6,74]. It is assumed that repetitive alveolar epithelial cell injury and subsequent aberrant repair lead to excessive growth factor activation and fibrotic transformation [6,73,74]. Multiple cytokines and growth factors, such as transforming growth factor (TGF)-β1, interleukin (IL) -4, -13, -21 [75] and angiotensin II (ANGII) [76,77] have been identified as potent regulators of fibrotic processes, directly or indirectly, for example, by regulation of the expression of coagulation factors which in turn contributes to the development of IPF [78–80].

The role of ANGII is well established in the pathogenesis of IPF. It was demonstrated that fibroblasts isolated from IPF lungs produce more ANGII as compared to fibroblasts isolated from donor lungs [81]. ANGII was found to induce apoptosis of epithelial and endothelial cells within lung tissue via ANGII receptor subtype AT(1) [82,83]. In addition, ANG II and aldosterone, a hormone regulated by the renin-angiotensin system, may significantly enhance the proliferation of various cells [84–86], suggesting that use of ANGII-receptor antagonists and aldosterone-receptor inhibitors may prevent fibrosis. Indeed, it was shown that a blockade of ANGII signaling attenuates the development of pulmonary and renal fibrosis [76,77,87], and, in contrast, administration of ANGII into the mouse potentiates perivascular and interstitial renal fibrosis, most likely via aggravation of PRMT1, which did not, however, alter ADMA levels in blood circulation [88]. This no net effect of ANGII treatment on systemic ADMA content could be explained by concomitant up-regulation of ADMA-generating (PRMT1) and ADMA-degrading (DDAH2) enzymes [88]. *In vitro* studies revealed that ANGII may induce PRMT1 expression and endothelial cell activation, resulting in the generation of reactive oxygen species (ROS) [89], key players in the establishment/progression of pulmonary fibrosis in animal models and possibly in human IPF [90,91]. Enhanced PRMT1 expression may also affect the regulation of transcription as PRMT1 was found to co-activate the nuclear factor-κB (NF-κB) signaling pathway [92] that, under stimulatory conditions, may trigger expression of MMP1 [93], MMP2 [94], MMP9 [93,95], collagen I [96], all proteins that have been reported to be up-regulated in the lungs of IPF patients and animal models of pulmonary fibrosis [6]. In addition, PRMT1 may induce structural changes in the target proteins, and thus influence the activation and differentiation of a variety of cells [21,97]. As renal and pulmonary fibrosis share similar features including fibrotic cytokines (ANGII, TGF-β) and ROS overproduction, and activated myofibroblasts originating from local mesenchymal cells, it can be speculated that enhanced PRMT1 expression may participate in the development of lung fibrosis by various mechanisms which may not necessarily have to be ADMA-dependent.

To date, there is only one report suggesting dysregulation of PRMT expression during lung fibrosis development. In this article, the authors examined the L-Arg metabolism in pulmonary fibrosis using a bleomycin-induced lung injury model [98]. In this model, fibrosis develops as a consequence of an overwhelming inflammatory response [99,100]. RNA and protein expression analysis of lung tissue of bleomycin-treated mice revealed dysregulation of expression of only one PRMT isoform, namely PRMT6. The PRMT6 level was found to be reduced during the inflammatory phase, but recovered afterwards, and it was upregulated in the fibrotic stage [98]. Unfortunately, the underlying molecular mechanism responsible for dysregulated PRMT6 expression in mouse fibrotic lungs was not investigated in this study. How PRMT6 may contribute to the development of pulmonary fibrosis can currently only be speculated, however, it has previously been reported that PRMT6 may regulate expression of genes, either by modulation of histone 3 function or by interaction with specific gene promoter regions [51,101]. One of the transcriptional targets of PRMT6 is thrombospondin-1 (TSP-1), a molecule critical for normal lung homeostasis [102]. Taking into consideration that TSP-1 is a major activator of TGF-β1 [103], a cytokine involved in the pathogenesis of pulmonary fibrosis [74,99], it is conceivable that altered PRMT6 expression might be an important factor in regulating the fibrotic processes. However, the mechanistic insights of PRMT action and causal relationship between fibrogenesis and PRMT expression and activity remain to be determined in future studies.

### 3.3. Pulmonary Hypertension

Pulmonary Hypertension (PH) is a hemodynamic and pathophysiological state defined by an increase in mean pulmonary artery pressure ≥25 mmHg. The current clinical classification comprises six clinical groups including pulmonary arterial hypertension (PAH; idiopathic; heritable; drug-induced; or associated with other disease conditions), PH due to left heart failure, due to lung diseases and/or hypoxia, and chronic thrombembolic PH (CTEPH). The pathophysiology of PH includes endothelial dysfunction and pulmonary arterial smooth muscle cell (PASMC) hypertrophy and proliferation, leading to the occlusion of pulmonary arterioles [104–107]. The mechanisms of endothelial vasodilator dysfunction in PH are multifactorial involving multiple pathways and mediators. One of them is NO, a well-known vasodilator that essentially controls a diverse range of pulmonary functions, such as macrophage activity, pulmonary artery vasodilation, or bronchoconstriction [108]. A product of protein arginine methylation, ADMA, is a naturally occurring nitric oxide synthase (NOS) inhibitor [49]. Elevated ADMA concentrations have been detected in the plasma of patients with idiopathic (i) PAH [109–111], CTEPH [112], PAH-related sickle cell disease [113] and systemic sclerosis [114], suggesting a strong association of circulating dimethylarginine levels with PH pathogenesis. It remains unclear which DDAH or PRMT isoforms control ADMA tissue and plasma levels under those pathological conditions. While some groups have reported decreased DDAH1 expression along with increased ADMA levels [115,116], others have detected decreased DDAH2 in the same conditions [110,117]. PRMT2 protein expression was found to be upregulated in mice exposed to chronic hypoxia, resulting in increased ADMA levels, thereby supporting an important role of PRMT-mediated ADMA generation in hypoxia-induced PH [34]. Comprehensive analysis of methylarginine content in the lungs of patients with PAH demonstrated significantly lower levels of protein-incorporated ADMA in the PAH lung tissue samples as compared to controls [118]. This was most likely a consequence of decreased expression of PRMT1 and asymmetrically dimethylated proteins. Moreover, *in vitro* and *ex vivo* studies have revealed that decreased PRMT1 expression directly leads to reduced cellular methylation and increased PASMC proliferation, which in turn might initiate, perpetuate, or potentate the vascular remodeling process in pulmonary arterioles in PAH [118].

### 3.4. Chronic Obstructive Pulmonary Disease and Asthma

Asthma is a chronic inflammatory disorder of the airways characterized by variable and usually reversible airflow obstruction. Asthma often has its first manifestation in childhood and may be classified as allergic (extrinsic) or non-allergic (intrinsic). In contrast, Chronic Obstructive Pulmonary Disease (COPD) is usually related to tobacco smoking and develops in mid to later life and is characterized by incomplete reversible airflow limitation resulting in a progressive decline in lung function leading to premature death. In both diseases inflammation plays an important role [119–121]. In asthma, airway inflammation is a multicellular process principally involving antigen presenting cells, eosinophils, mast cells and TH2 lymphocytes [121]. It was previously reported that activation of T lymphocytes is controlled by protein arginine methylation [13,122]. In this regard, methylation of the nuclear factor of activated T-cells (NFAT) interacting protein 45 (NIP45) by PRMT1 increases the activity of NFAT, which is required for the production of cytokines by T lymphocytes [123]. Similarly, PRMT1-dependent methylation of STAT proteins potentiates cytokine expression in various cells and thus impacts on many intracellular signaling pathways [124]. These findings suggest that targeting protein arginine methylation is a feasible strategy for modulation of the T lymphocyte function, offering a novel therapeutical option for the treatment of T cell-mediated disorders, such as autoimmune disease, transplant rejection, but also potentially COPD and asthma [13,122,125].

Enhanced ADMA levels have been demonstrated in allergically inflamed mouse lungs, as well as in lung and sputum samples of asthma patients [126,127]. In line with these findings exogenous administration of ADMA potentiated lung inflammation in a murine model of allergic asthma [128]. The altered ADMA metabolism in the previously mentioned pathological conditions might be a consequence of enhanced type I PRMT expression or activity. Indeed, increased expression of PRMT2 has been demonstrated in a murine model of lung allergic airway inflammation [126] and elevated levels of PRMT1, PRMT2, PRMT3 (but not PRMT4) were found in the lungs of a rat model of Ag-induced pulmonary inflammation (AIPI) [129]. In AIPI rats, epithelial IL-4-triggered PRMT1 expression enhanced eotaxin-1 and CCR3 receptor expression thus promoting recruitment of eosinophils to the lung. Administration of AMI-1, specific inhibitor of PRMT activity, to AIPI rats ameliorated pulmonary inflammation, reduced humoral immune response and abrogated eosinophil infiltration suggesting a pivotal role of PRMT, particularly PRMT1, in asthma pathogenesis [129].

As cigarette smoke represents the main risk factor for COPD, several studies have investigated the relationship between cigarette smoking and ADMA levels. While some studies have found decreased ADMA levels in smokers as compared to non-smokers, others have detected increased amounts of ADMA in cigarette smoking subjects [130,131]. Despite conflicting results on arginine metabolism in COPD patients, altered ADMA levels in smokers might be associated with dysregulated PRMT activities and the ubiquitin-proteasome system, an ATP-dependent proteolytic pathway, which has been implicated in the ADMA metabolism [36,37].

In summary, since limited data on protein arginine methylation in COPD and asthma exists, further *in vitro* and *in vivo* studies are needed to decipher the relationship between dysregulated PRMT expression and/or activity and pathogenesis of the aforementioned diseases.

## 4. Conclusions

**Figure 2:**
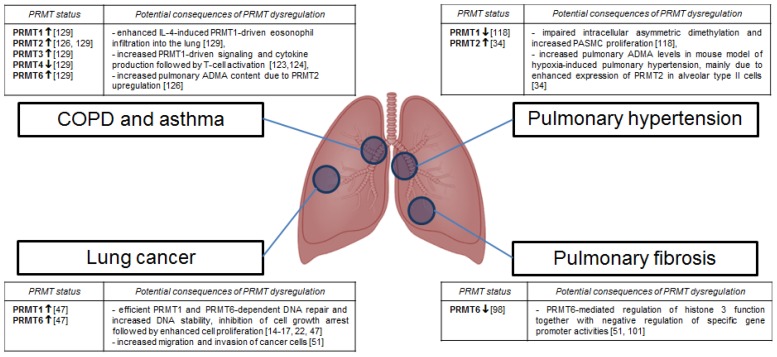
Dysregulation of PRMTs in human and experimental pulmonary diseases. Members of the PRMT family and potential mechanisms of their action in the development and progression of pulmonary diseases (PD) are indicated. Arrows demonstrate PRMTs expression status, which has previously been reported in the literature. ↑, enhanced expression; ↓, impaired expression.

In conclusion, dysregulated protein arginine methylation and abnormal expression of PRMTs have recently been shown to contribute to the pathogenesis of PD in experimental animal models as well as in humans (Figure 2). However, it is still not clear how and to what extent PRMT-dependent posttranslational modification of proteins may influence protein functions and how dimethylarginines can regulate biological processes. It seems that PRMT dependent alterations of cellular activities might be the cause or the result of pathological changes observed in discussed PD. Therefore, future studies are required to answer the question as to whether PRMTs may offer a new potential therapeutical option for the treatment of PD.
